# Microplastic transport in soil by earthworms

**DOI:** 10.1038/s41598-017-01594-7

**Published:** 2017-05-02

**Authors:** Matthias C. Rillig, Lisa Ziersch, Stefan Hempel

**Affiliations:** 1 Freie Universität Berlin, Institut für Biologie – Plant Ecology, Altensteinstr. 6, D- 14195 Berlin, Germany; 2 Berlin-Brandenburg Institute of Advanced Biodiversity Research (BBIB), D- 14195 Berlin, Germany

## Abstract

Despite great general benefits derived from plastic use, accumulation of plastic material in ecosystems, and especially microplastic, is becoming an increasing environmental concern. Microplastic has been extensively studied in aquatic environments, with very few studies focusing on soils. We here tested the idea that microplastic particles (polyethylene beads) could be transported from the soil surface down the soil profile via earthworms. We used *Lumbricus terrestris* L., an anecic earthworm species, in a factorial greenhouse experiment with four different microplastic sizes. Presence of earthworms greatly increased the presence of microplastic particles at depth (we examined 3 soil layers, each 3.5 cm deep), with smaller PE microbeads having been transported downward to a greater extent. Our study clearly shows that earthworms can be significant transport agents of microplastics in soils, incorporating this material into soil, likely via casts, burrows (affecting soil hydraulics), egestion and adherence to the earthworm exterior. This movement has potential consequences for exposure of other soil biota to microplastics, for the residence times of microplastic at greater depth, and for the possible eventual arrival of microplastics in the groundwater.

## Introduction

Plastic, as a versatile, inexpensive and resistant material for a huge variety of products has without a doubt revolutionized our daily lives^1, 2^. However, even though benefits to society are enormous^3^, plastic has also developed into a serious environmental problem^1, 4^. In particular, there has been increasing awareness of the environmental problems caused by the accumulation of microplastic in the marine environment^5, 6^. Microplastic forms by degradation of larger plastic pieces, or is already industrially produced as material of this small size. It is often considered to be material <5 mm, but particles frequently are in the range of several micrometers only^1^. The different size gives rise to properties quite different from that of environmental contamination by larger plastic pieces^5^. First, due to their small size they can be ingested^5^, and they may thus accumulate along the food chain. Secondly, the particles possess a large surface area, and pollutants may be sorbed to these surfaces^5^.

While research on microplastic distribution and effects is well underway in the marine setting, there is a dearth of information regarding terrestrial and soil environments^7, 8^, even though there are good reasons to assume that this material is present at a broad scale^8^: plastic is ever-present in our lives and our cities, as well as other human-influenced landscapes^9^. In fact, the presence of larger pieces of plastic in soils with anthropogenic influence is nothing new; this can be a common trait in urban soils or Technosols. Occurrence of *bona fide* microplastic particles in soil, however, has not been frequently documented. Zubris and Richards^9^ found synthetic fibers using polarized light microscopy in several soils in the U.S. to which organic wastewater sludge had been applied. In field sites, such fibers were found 15 years after last application, highlighting the potential persistence of this material in the soil environment. The same study also found evidence for downward movement of fibers by unknown agents, suggesting that various soil horizons may be affected. More recently, Fuller and Gautam^10^, using pressurized fluid extraction combined with FTIR spectral matching, could quantify microplastic in various soils from an industrial area in Australia, adding to the growing body of evidence of microplastic presence in the terrestrial environment. Nizzetto *et al*.^11^ even estimate that annually up to 700,000 tons of microplastic may enter farmland via manure application in Europe and North America, more than the burden of marine surface waters.

Microplastics are thus likely widespread and, irrespective of their origin, are expected to arrive at the soil surface. Since microplastic could have potential adverse effects on soil biota, for example earthworms^12^, a critical research need relates to the incorporation of this material into the soil profile. Only when this material is transported into the soil can it be expected to lead to broad exposure of soil biota to these particles. Here we explored potential transport of surface-deposited microplastic particles of different sizes by the activity of anecic earthworms. Anecic earthworms are excellent soil biota with which to conduct these tests, since they potentially can physically move particles into the soil^13^, and they are also well-known for altering soil hydraulic properties (biopore formation)^14^ in a way that could promote microplastic downward movement. Previously, Huerta Lwanga *et al*.^15^ have shown that *Lumbricus terrestris* L. could in fact incorporate microplastic particles into burrows.

## Materials and Methods

### Earthworms, soil and microplastic

For these experiments we used the anecic earthworm *Lumbricus terrestris* L. (Oligochaeta, Lumbricidae; obtained from Wurmwelten, Dassel, Germany), a species native to Europe that is frequently used in lab and field trials (e.g. refs 12, 16 and 17). In the experiment we used four adult, healthy earthworms with a body weight between 3 to 5 g each per experimental unit; the individuals were carefully washed, dried on filter paper and weighed prior to their addition to the experimental pots.

As experimental soil we used material from the top horizon of an Albic Luvisol (73.6% sand, 18.8% silt and 7.6% clay; 6.9 mg/100 g P; 5.0 mg/100 g K; pH 7.3)^18^, which was field collected from a meadow in Berlin, sieved (5 cm) to remove stones or debris, and then steamed (90 °C, 2 h) to eliminate other soil biota that could move microplastic.

For this study we used clear, approximately spherical polyethylene (PE) microplastics (Cospheric, Santa Barbara, CA, USA), containing no additives or solvents (density 0.96 g cm^−3^). PE is among the major plastic types that are used worldwide^3^. We assumed, based on a previous study^12^, which also used PE, that earthworms might ingest and/or transport microparticles of different sizes to a different extent. For this reason, we used four commercially available different particle size ranges: 710–850 μm (PE-1), 1180–1400 μm (PE-2), 1700–2000 μm (PE-3) and 2360–2800 μm (PE-4). All particles were white in appearance, facilitating their retrieval from soil.

### Experimental setup

We carried out a fully factorial experiment in which each added PE-microplastic size was combined with earthworm presence/absence (n = 5), for a total of 40 pots. Additionally, there was a control without microplastics, but with earthworms (n = 10) to ascertain any effects on earthworms. This 21-day experiment was carried out in an air-conditioned greenhouse with a temperature of 20 °C (±2 °C). As containers (experimental units) we used plant pots (volume: 3 L; height: 19.2 cm; diameter: 17.0 cm) for which the bottom was sealed with permeable black fleece in order to avoid standing water and to prevent the earthworms from escaping during the experiment. This setup was not designed to ascertain the maximum depth to which earthworms could move particles, as this earthworm species can produce burrows to >0.3 m depth^14^, but to clearly demonstrate the movement of particles. Preliminary trials had shown that earthworms can be reared in containers of this size. All containers were filled with 2.5 kg of soil, and all containers received 5.0 g of dried *Populus* spp. leaf litter (suitable material for these earthworms)^12^, added as chopped material to the soil surface, to provide a sufficient amount of organic matter for the earthworms. We did not monitor soil water content, but all pots were watered with 100 mL of water at the same time of day every 2 d (starting 10 d before earthworm addition), which was found suitable in preliminary trials. Pots could freely drain during the study. Microplastic particles were added to the soil surface by weight (750 mg of the differently sized PE-microplastic particles) at the beginning of the experiment. This amount of microplastic did not have overt toxic effects on earthworms in preliminary trials. For the different sizes this translated to 2625 particles (PE-1), 424 particles (PE-2), 203 particles (PE-3) and 75 particles (PE-4).

### Harvest and measurements

At the end of the experiment (21 d), the remaining leaf litter and the earthworm casts (around the earthworm burrows) were collected from the soil surfaces. The visible microplastic particles at the soil surfaces were collected and counted, and any presence of microplastic in surface middens was noted. Earthworms were then extracted by hand: this occurred during carefully extracting cores for microplastics (see below), if earthworms were encountered, and all remaining earthworms were extracted after the coring for microplastics was completed. All earthworms were washed, carefully dried off on paper towels, weighed and placed into empty, moistened Petri dishes. The earthworms were kept for 36 h to ensure that the earthworms empty their guts completely, and to search for any microplastic particles in the casts, since the species *L*. *terrestris* needs around 20 h to fully empty their gut^19^. At the end of this period, any presence of microplastic particles in casts was noted.

We approached the analysis of microplastic in soil by using soil cores to sample the experimental units to three depths. In each pot we took two soil cores (diameter: 40 mm), taken to avoid spots with visible earthworm burrows but otherwise randomly. We sliced these cores into three equal portions of 3.5 cm (top: 0–3.5 cm, middle: 3.5–7.0 cm, and bottom: 7.0–10.5 cm), and dried the soils at 40 °C for 48 h before further processing. We then used an aqueous extraction/flotation method to extract the microplastic particles, exploiting their low density (0.96 g cm^−3^). Briefly, soil samples were suspended in 25 mL water, the suspension was then vortexed (10 s), centrifuged (Thermo Scientific Heraeus Multifuge, 2500 rpm, 5 min, 21 °C) and decanted through a series of sieves. Microplastic particles were collected on a sieve (250 μm) and then counted. We did not formally assess efficiency of this extraction method, since it was not necessary for our question, but re-extractions of soils did not yield any further microplastic particles. We expressed data as relative counts of microplastic particles. This was done either as a percentage of particles added for particles remaining on the surface (i.e. number of particles retrieved from the pot surface/number of particles added to the pot), or as a percentage of particles retrieved in the cores for the depth distribution (i.e. number of particles extracted from the respective layer in the pot/number of particles extracted from all layers in the pot) in order to compare among the different particle sizes.

### Statistical analyses

We separated the downward movement of microplastic particles into two components, reflecting the measurements at harvest time: (a) the disappearance of material from the soil surface and (b) the vertical distribution of these transported particles in the soil profile. For the first component, we applied a linear model to test the effects of earthworm presence, microplastic particle size and their interaction on the relative proportion of particles recovered from the surface of the pots at the end of the experiment. For the latter component, we used a linear mixed effects model with layer, earthworm presence and microplastic particle size as fixed effects and pot id as random effect to account for the repeated analysis of the same pot in different layers.

Data on earthworm mortality and change in body mass during the experiment were analyzed by a generalized linear model with binomial error distribution and linear model, respectively in both cases with the type of microplastic as predictor variable.

Model assumptions for all models were validated using diagnostic plots of residuals. All analyses were conducted in R version 3.3.0^20^ with the R packages nlme^21^ and sciplot^22^ and all data are provided in the Supplementary Information.

## Results

**Figure 1:**
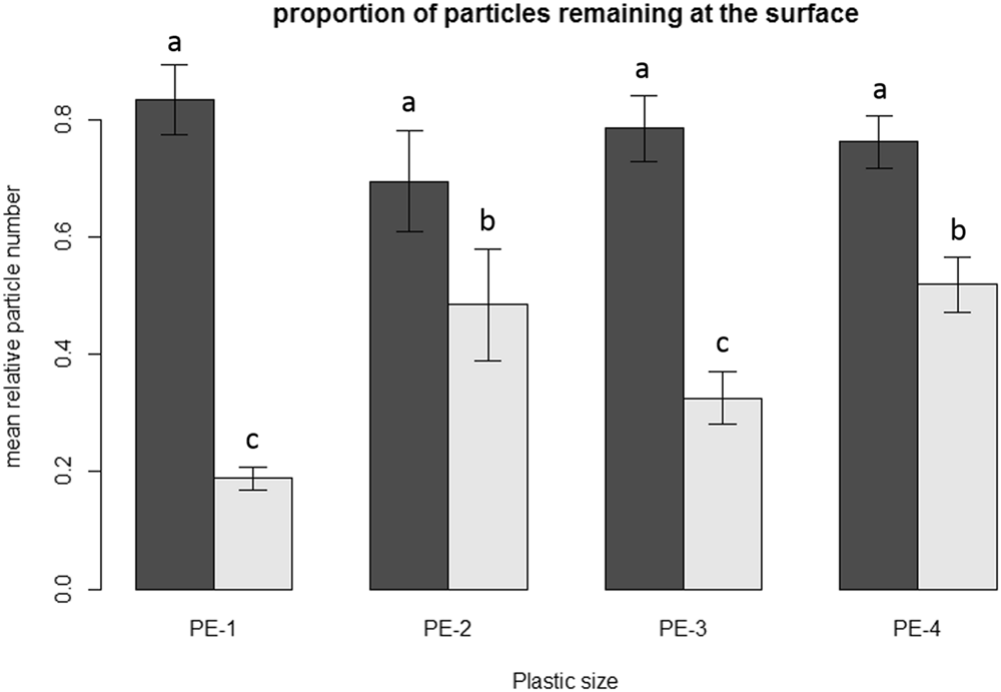
Mean relative microplastic particle numbers recovered from the soil surface after the experiment (expressed as a percentage of the number of particles added to the soil surface at the beginning), error bars indicate standard error. Dark grey bars show values in absence of earthworms, while light grey bars give data with earthworms. Results are shown for four different particle size ranges: 710–850 μm (PE-1), 1180–1400 μm (PE-2), 1700–2000 μm (PE-3) and 2360–2800 μm (PE-4). Different letters above bars indicate significant differences (P < 0.05; Tukey-Kramer HSD).

The presence of earthworms had a significant positive effect of microplastic particle transport away from the soil surface (P < 0.001, see Fig. 1), and the magnitude of this effect was significantly modified by microplastic size (P = 0.03).

**Figure 2:**
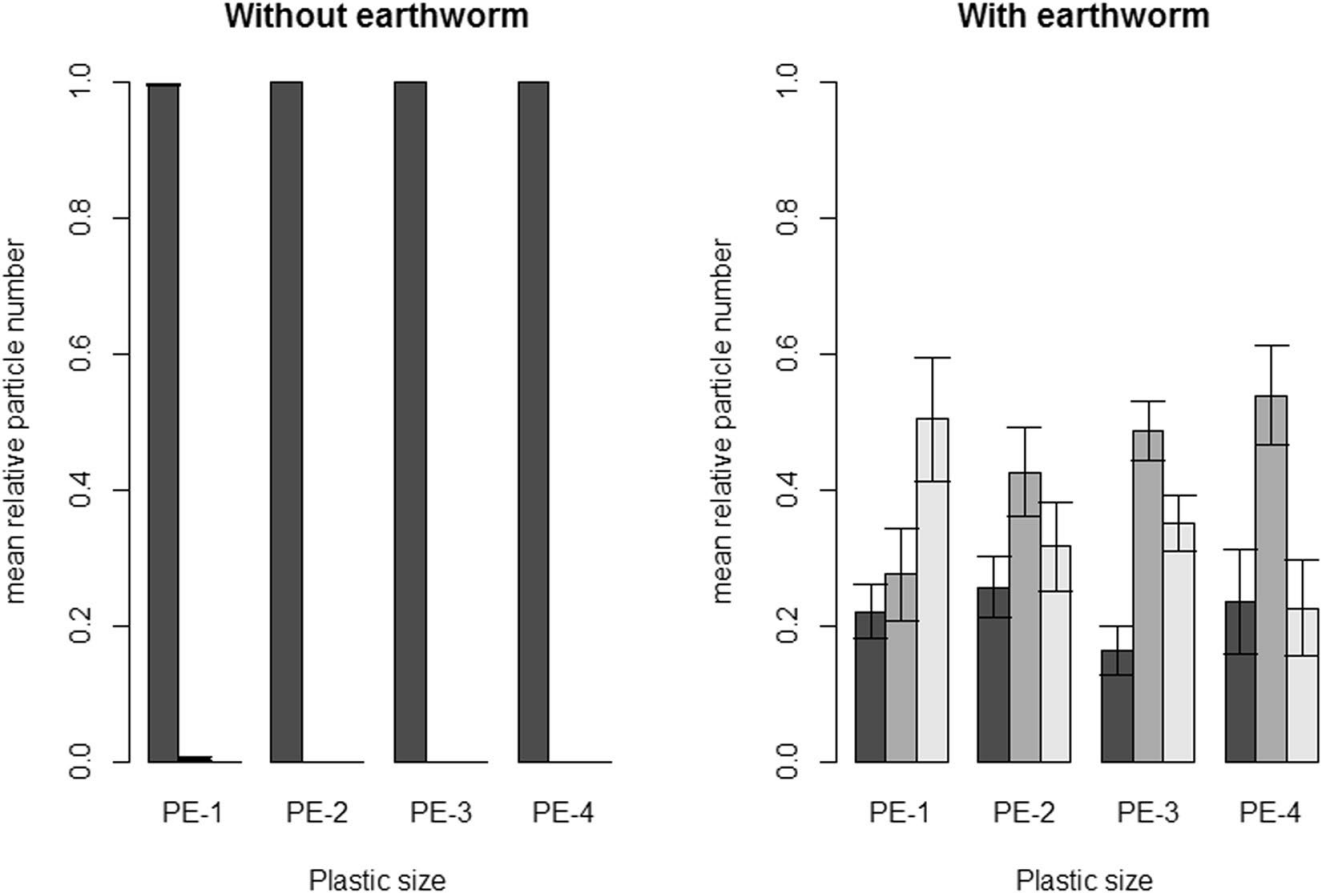
Distribution of the mean relative microplastic particle numbers (expressed as a percentage of all particles retrieved in the cores per pot) among the different soil layers, error bars indicate standard errors. The left panel shows data in the absence of earthworms, the right panel with earthworms. Dark grey bars show data for the top soil layer, while medium and light grey bars show data for the middle and bottom layer, respectively. Results are shown for four different particle size ranges: 710–850 μm (PE-1), 1180–1400 μm (PE-2), 1700–2000 μm (PE-3) and 2360–2800 μm (PE-4).

There was also a clear overall effect on microplastic distribution in different soil depths as a function of earthworm presence and particle size (both P < 0.001, Fig. 2). Without earthworms microplastic particles stayed in the top soil layer, while with earthworms microplastic particles of all sizes reached the middle and bottom soil layers within the 21 d experimental period, with the smallest particles (PE-1) having been moved most into the bottom soil layer (interactive effect of earthworm presence, particle size and layer, P < 0.001). The other three particle sizes were mostly found in the middle layer in the presence of earthworms.

**Figure 3:**
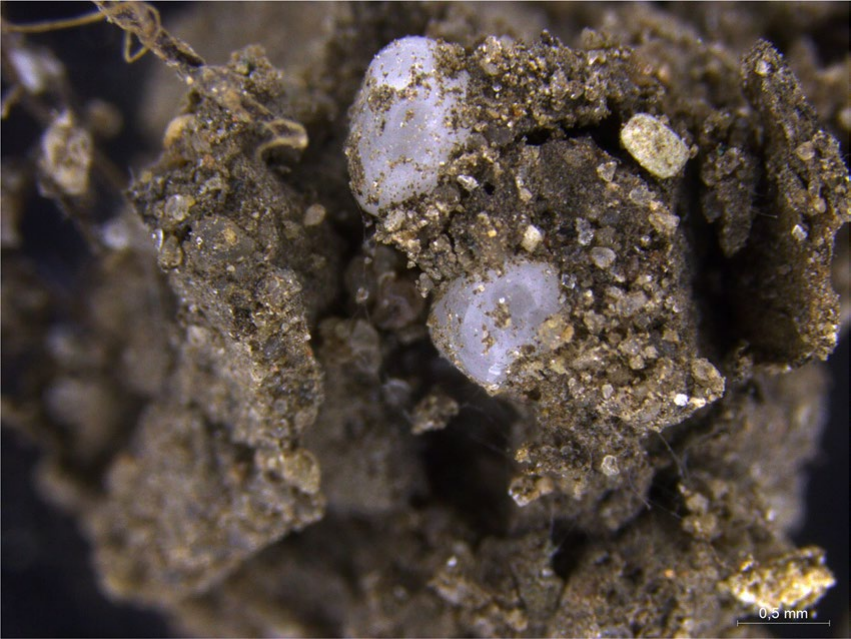
Polyethylene (PE-2; 1180–1400 μm) microplastic particles incorporated into surface middens. Picture taken during the harvest of the experiment.

**Figure 4:**
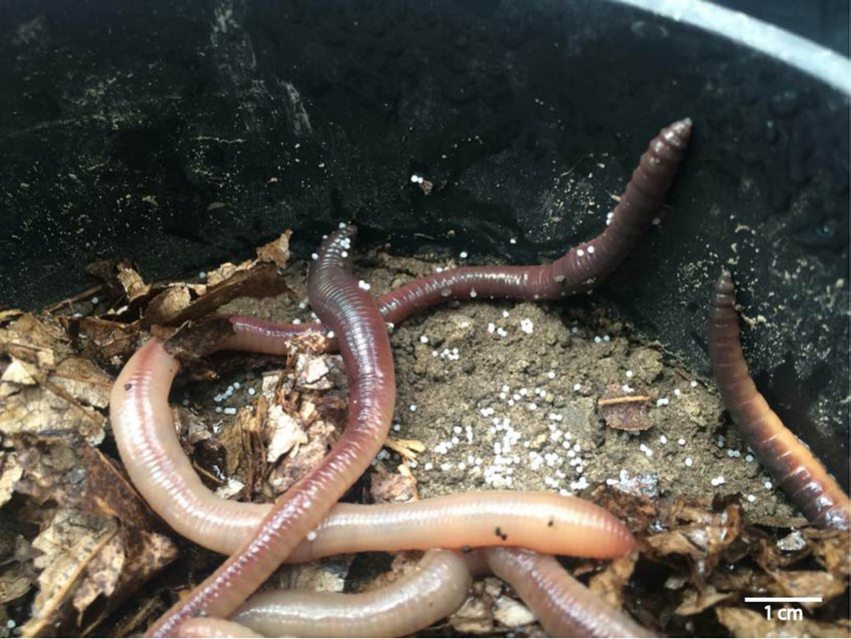
Polyethylene (PE-1; 710–850 μm) microplastic particles adhering to the skin of two earthworms. Picture taken during the harvest of the experiment.

Microplastic particles were present in casts (observations in Petri dishes), but only for the two smaller size classes; no microplastic particles were observed in the casts for the two larger size classes. There were microplastic particles present in surface middens (observations in the pots) in all microplastic size treatments with earthworms (Fig. 3). We also observed microplastic beads adhering to the earthworm body (Fig. 4).

Earthworm mortality occurred during the experiment across all treatments (mean 33.3% ± 6.7% over all pots). Mortality effects of microplastic could not be detected (P > 0.5, data not shown). The same was true for effects on earthworm weights, which generally decreased from an average 4.1 g ± 0.02 g (standard error) before to an average of 3.4 g ± 0.3 g after the experiment, without detectable differences between pots with or without microplastic particles (P > 0.5, data not shown).

## Discussion

We showed, as one of the first studies of this type^15^, that PE microplastic particles can clearly be transported relatively quickly by anecic earthworms downward into a soil profile from the surface to a depth of 10 cm, where the number of particles transported appears to be influenced by particle size: the smallest particles were found most in the deepest layer. Our approach here was to use a controlled experiment in pots in the greenhouse with surface-added microplastic particles, which could afterwards be retrieved from the experimental soils at various depths. There was a clear expectation for earthworms to contribute to vertical transport of microplastics, since earthworms have previously been shown to be able to transport other particles, typically of several mm size, including plant seeds (e.g., ref. 23) or sand particles^24^. Earthworms have also been implicated in the vertical transport of biochar particles in the field^25^. Additionally, *Lumbricus terrestris* was also recently shown to incorporate PE microplastic particles in its burrows during a 14-day mesocosm study^15^.

The exact transport mechanisms in this and other studies on particle transport are typically not known, but are thought to include attachment to the outside of the earthworm (as we often observed PE particles sticking to the earthworms), movement down the burrows with water, casting activity, and movement by the earthworm following passage through the gut (the latter is supported by the pervasive presence in droppings). To further support the ingestion/egestion mechanism, Huerta Lwanga *et al*.^12^ have also documented the presence of PE microplastic particles (<150 μm size was used in their study) in earthworm egestate. Our study was not specifically designed to distinguish the relative importance of these transport mechanisms. Now that transport by earthworms has been shown, future studies should aim at disentangling these different transport pathways, also as a function of soil properties (in particular texture and structure) and amount of plastic material. For example, Huerta Lwanga *et al*.^15^, having compared different concentrations of PE microplastic (rather than sizes, as we have done), find concentration-dependent incorporation of microplastic material into burrow walls (data normalized to worm biomass). Additionally, it will be important to next design studies under field conditions to capture transfer rates under more realistic conditions.

Earthworms are not the only soil biota producing biopores, for example also plant roots produce extensive biopores (e.g., ref. 16). This means that also other soil biota could be examined for the potential to enhance movement of microplastic down the soil profile. In addition to biopore- producing biota, other soil biota may also contribute to the vertical and horizontal movement. It is conceivable that termites, collembola, enchytraeids or nematodes move such particles in soil^13^, albeit likely at a smaller spatial scale than what we observed for earthworms in the short term. At a larger scale, animals such as gophers, moles, or voles may be important. In agroecosystems, where microplastic application is perhaps most likely, there additionally is the factor of ploughing, which would be expected to greatly contribute to incorporation of materials into the soil.

There are several possible implications of microplastic transport down the soil profile: (i) Decomposition of organic material is generally much slower deeper in the soil (e.g., ref. 26), where microbial populations are much reduced^27^; this would mean that microplastic, which is slow to turn over in the environment in the first place, may have even longer persistence once it reaches greater depths in the soil profile. This also highlights that as methods for quantifying microplastics in soil are being developed^10^, not only should surface soils be tested, but also subsoils. It is presently unknown if microplastic makes significant contributions to soil organic carbon pools at various soil depths. (ii) Microplastic could potentially also reach groundwater, after passage through the soil profile, where it may then lead to undesirable effects akin to those already extensively documented in other aquatic environments^5^. (iii) Microplastic that arrives in the soil may be subject to further disintegration, leading to the production of nano-sized material, which could have different functional properties and pose different environmental risks.

In conclusion, our study showed the movement of PE microplastic particles into soil by the action of soil animals, with the smallest particles moving proportionally the most. Given the likely arrival of microplastics at the soil surface this suggests that soil biota along the profile will be exposed to these particles, highlighting the need to further examine their effects and fate in the terrestrial environment.

## Electronic supplementary material

Dataset 1
